# An Adaptive Imitation Learning Framework for Robotic Complex Contact-Rich Insertion Tasks

**DOI:** 10.3389/frobt.2021.777363

**Published:** 2022-01-11

**Authors:** Yan Wang, Cristian C. Beltran-Hernandez, Weiwei Wan, Kensuke Harada

**Affiliations:** ^1^ Department of Systems Innovation, Graduate School of Engineering Science, Osaka University, Suita, Japan; ^2^ Automation Research Team, Artificial Intelligence Research Center, National Institute of Advanced Industrial Science and Technology, Tsukuba, Japan

**Keywords:** compliance control, imitation learning, reinforcement learning, robotic assembly, robot autonomy

## Abstract

Complex contact-rich insertion is a ubiquitous robotic manipulation skill and usually involves nonlinear and low-clearance insertion trajectories as well as varying force requirements. A hybrid trajectory and force learning framework can be utilized to generate high-quality trajectories by imitation learning and find suitable force control policies efficiently by reinforcement learning. However, with the mentioned approach, many human demonstrations are necessary to learn several tasks even when those tasks require topologically similar trajectories. Therefore, to reduce human repetitive teaching efforts for new tasks, we present an adaptive imitation framework for robot manipulation. The main contribution of this work is the development of a framework that introduces dynamic movement primitives into a hybrid trajectory and force learning framework to learn a specific class of complex contact-rich insertion tasks based on the trajectory profile of a single task instance belonging to the task class. Through experimental evaluations, we validate that the proposed framework is sample efficient, safer, and generalizes better at learning complex contact-rich insertion tasks on both simulation environments and on real hardware.

## 1 Introduction

Contact-rich insertion is a ubiquitous robotic manipulation skill in both product assembly and home scenarios. Some contact-rich insertion tasks involve nonlinear and low-clearance insertion trajectories and require varying force control policies at different phases, which we define as *complex* contact-rich insertion tasks, such as ring-shaped elastic part assembly and USB insertion. Such tasks demand skillful maneuvering and control, which makes them challenging for robots.

Imitation learning (IL) is a promising approach to tackle complex contact-rich insertion tasks by reproducing the trajectory and force profiles from human demonstrations. However, there are some *concerns* that prevent IL from working efficiently and safely in actual applications:

1) Force profiles are not easy to acquire from demonstrations compared with trajectory profiles: Trajectory profiles can be easily obtained from kinesthetic teaching, teleoperation, simulation, among other methods, but force profiles usually demand additional haptic devices (Kormushev et al., 2011). Even with the force sensor that is integrated into the robot, it suffers from the strict position limit, i.e., the hand of the demonstrator should never be between the end-effector (EEF) and the force sensor, which usually makes the demonstrations of the complex contact-rich tasks inconvenient. Also, when there is no real robot available, force profiles from simulated environments can be unsuitable for actual tasks due to the reality gap (Mirletz et al., 2015).
2) Motion shift of the EEF of the manipulator exacerbates the compounding error problem (Ross and Bagnell, 2010; Ross et al., 2011) of IL because IL usually learns a one-step model that takes a state and an action and outputs the next state, and one-step prediction errors can get magnified and lead to unacceptable inaccuracy (Asadi et al., 2019).
3) Demonstrations are usually task specific and require human repetitive teaching efforts for new tasks even if demonstrations with topologically similar trajectories have already been collected.

For the *first* concern, the lack of proper force profiles in human demonstrations can be complemented by model-free reinforcement learning (RL), which is an effective method to handle contact-rich insertion tasks (Inoue et al., 2017; Vecerik et al., 2019) by interacting with the environment. Beltran-Hernandez et al. (2020) presents an RL-based control framework for learning low-level force control policies to enable rigid position-controlled robot manipulators to perform contact-rich tasks. However, the exploratory nature of RL can lead to low-quality trajectories for complex contact-rich insertion tasks, which causes hardware wear and tear or even damage due to a myriad of collisions during the training process on a real robot. Therefore, high-level control policies, which can give proper commands of nominal trajectories of the EEF of the manipulator are necessary to alleviate the situation. We define the high-level control policy which provides the nominal trajectory as the *skill* policy, and the low-level control policy which generates the specific parameters of the controller as the *motion* policy. A skill policy can be learned by IL, but as the *second* concern above presents, the motion drift of EEF away from the demonstrated trajectory usually occurs during a task. Therefore, we proposed a novel hierarchical goal-conditioned IL (HGCIL) method (Wang et al., 2021) to learn the skill policy to facilitate the EEF to recover from deviate poses through self-supervised learning. Finally, as the *last* concern states, there are situations where a human has to demonstrate a set of tasks with topologically similar trajectories. These tasks differ from each other in terms of geometric characteristics such as size or shape of the work-pieces. Therefore, we seek to generalize an existing trajectory profile to its variations so that human efforts on new demonstrations can be reduced. Dynamical movement primitives (DMPs) (Ijspeert et al., 2013) model is a typical dynamic system-based technique that has been widely applied in the field of IL for encoding both periodic and discrete motion data. DMPs can generate a trajectory or control signal that can be flexibly adjusted to guide the real system without manual parameter tuning or affecting the overall convergence and stability. They can also be modulated to meet different requirements, such as obstacle avoidance (Park et al., 2008; Hoffmann et al., 2009), by adding feedback terms. Therefore, we consider using DMPs to adapt an existing trajectory profile to new tasks so that we can learn new control policies based on the generalized trajectory profiles.

**FIGURE 1:**
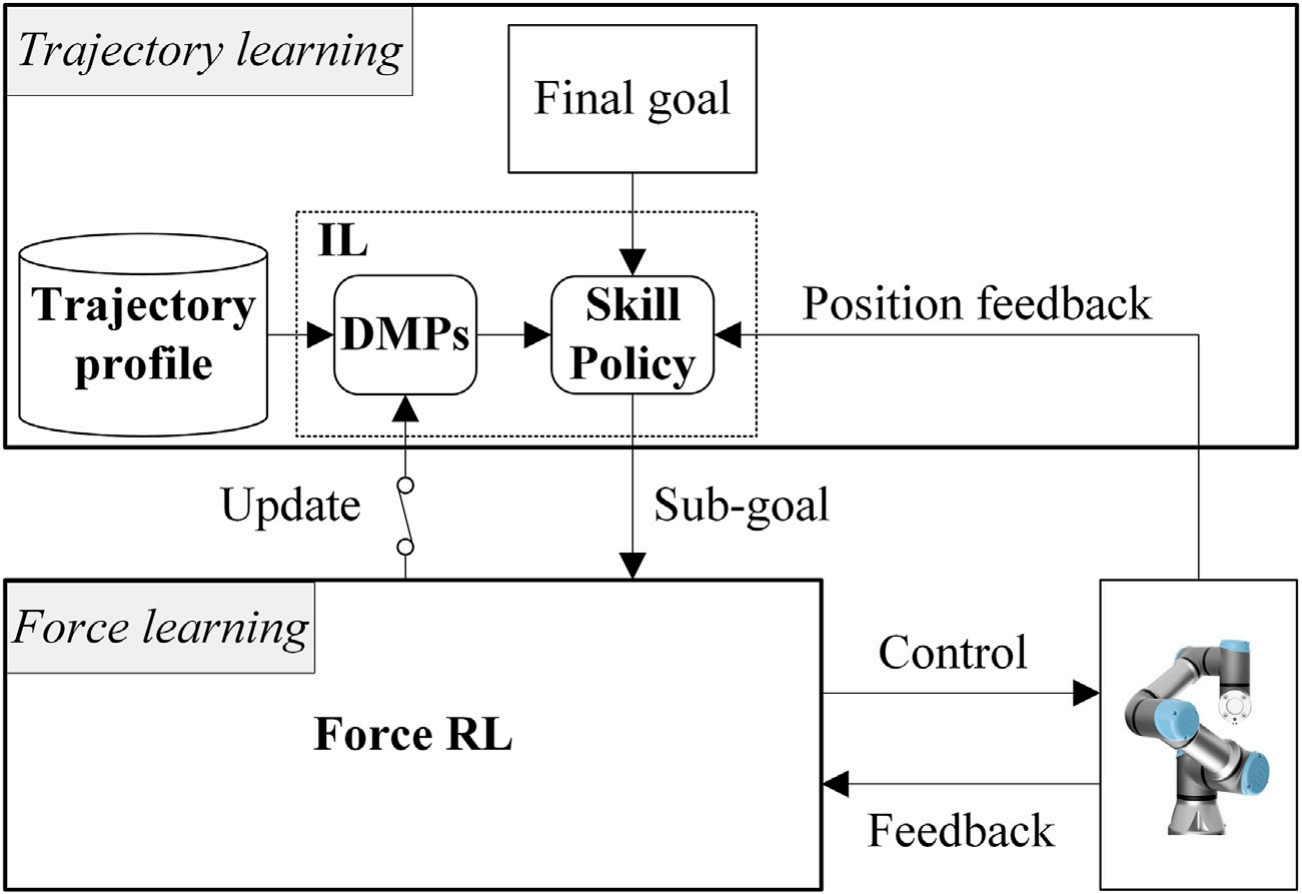
System overview of the adaptive robotic imitation framework. The upper and the lower part are the trajectory learning and the force learning parts, respectively. The switch symbol between the reinforcement learning (RL) agent and the dynamical movement primitives (DMPs) module means the update of DMPs is executed using a modular learning strategy.

The main contribution of this paper is the development of an adaptive imitation learning framework for robot manipulation (Figure 1), which introduces DMPs into a hybrid trajectory and force-learning framework in a modular fashion, to learn the control policies of a specific class of complex contact-rich insertion tasks based on the trajectory profile of a single instance (note that a trajectory profile can include several trajectory demonstrations of a task instance), thus, relieving human demonstration burdens. We show that the proposed framework is sample efficient, generalized to novel tasks, and is safe enough to be qualified for the learning on both simulated environment and real hardware.

The rest of this paper is organized as follows: After discussing the most related work in the *Related work* section, we set up our problem and introduce some techniques applied in our framework in the *Preliminaries* section. In the *Adaptive robotic imitation framework* section, we describe the overview and details of the proposed adaptive imitation learning framework. Then we experimentally evaluate the performance of this framework on simulated environment and real hardware using a UR3e robotic arm in the *Experimental evaluation* section.

## 2 Related work

In this section, we provide an overview of the application of IL and RL approaches in the context of contact-rich insertion tasks and the position of our work in the existing literature.

### 2.1 Imitation learning

Imitation learning (IL), also referred to as learning from demonstration (LfD), is a powerful approach for complex manipulation tasks, which perceives and reproduces human movements without the need of explicit programming of behavior (Takamatsu et al., 2007; Kormushev et al., 2011; Suomalainen and Kyrki, 2017; Hu et al., 2020). Among the IL approaches, DMPs (Ijspeert et al., 2013) have shown the ability to generalize demonstrations in different manipulation tasks (Peters and Schaal, 2008; Metzen et al., 2014; Hu et al., 2018; Sutanto et al., 2018). However, the forces and torques that a human applies during the demonstrations of contact-rich tasks are required to regress a proper admittance gain of robot controller (Tang et al., 2016) or to match with modified demonstrated trajectories using DMPs (Abu-Dakka et al., 2015; Savarimuthu et al., 2017). To quickly program new peg-in-hole tasks without analyzing the geometric and dynamic characteristics of workpieces (Abu-Dakka et al., 2014) exploits demonstrations and exception strategies to develop a general strategy that can be applied to the objects with similar shapes, which need to be inserted. However, force profiles are still essential for such strategies to modify the trajectories of the learned movements.

In contrast, we study the case wherein only the trajectory profile of a single instance is available in a class of complex contact-rich insertion tasks, and based on this trajectory profile, we manage to solve other variations of this instance with different object sizes or shapes but topologically similar insertion trajectories without explicitly knowing the concrete geometric characteristics. In this context, it does not help even if the original force profile is available because the new trajectories are unknown so that we cannot match the trajectory and the force profiles.

### 2.2 Reinforcement learning

Reinforcement learning (RL) methods have been widely used for contact-rich robotic assembly tasks (Inoue et al., 2017; Thomas et al., 2018; Vecerik et al., 2019; Beltran-Hernandez et al., 2020) to circumvent difficult and computationally expensive modeling of environments. However, sample efficiency and safety problem have always been issues that affect its practicality in complex contact-rich manipulation tasks.

To improve the sample efficiency and guarantee the safety of RL, human prior knowledge is usually incorporated for learning complex tasks. One such way is reward shaping (Ng et al., 1999), where additional rewards auxiliary to the real objective are included to guide the agent toward the desired behavior, e.g., providing punishment when a safety constraint such as collision is violated (Beltran-Hernandez et al., 2020). Generally, reward shaping is a very manual process. It is as difficult to recover a good policy with reward shaping as to specify the policy itself (Johannink et al., 2019). Although some prior work considers reward shaping as a part of the learning system (Daniel et al., 2015; Sadigh et al., 2017), human efforts are still necessary to rate the performance of the system. Therefore, another way occurs that human prior knowledge is included in RL through demonstration (Atkeson and Schaal, 1997) to guide the exploration. Some work initializes RL policies from demonstration for learning classical tasks such as cart-pole (Atkeson and Schaal, 1997), hitting a baseball (Peters and Schaal, 2008), and swing-up (Kober and Peters, 2009). Beyond initialization using demonstration, some promising approaches incorporate demonstrations with the RL process through replay buffer (Vecerik et al., 2017; Nair et al., 2018) and fine-tuning with augmented loss (Rajeswaran et al., 2018). However, these methods require humans to be able to teleoperate the robot to perform the task so that the observation and action spaces of demonstration (state–action pairs) are consistent with the RL agent, which is not always available for an industrial manipulator.

Considering the lack of teleoperation system, residual RL (Johannink et al., 2019) combines the conventional controller, which ships with most robots with deep RL to solve complex manipulation tasks, where the problems can be partially handled with conventional feedback control, e.g., with impedance control, and the residual part, including contacts and external object dynamics, is solved with RL. Based on Johannink et al. (2019), Davchev et al. (2020) proposes a residual LfD (rLfD) framework that bridges LfD and model-free RL through an adaptive residual learning policy operating alongside DMPs applied directly to the full pose of the robot to learn contact-rich insertion tasks. However, Davchev et al. (2020) does not discuss how to handle different force requirements at different phases, e.g., the search phase and insertion phase, of the insertion task.

In the proposed framework, we utilize DMPs on the skill level together with a novel HGCIL approach to provide nominal trajectories for the controller to follow and learn the motion policy of the controller by RL. Specifically, the framework learns the time–variant force–control gains to behave accordingly at different phases of the insertion task, which is not discussed in Davchev et al. (2020), and DMPs are also updated by RL to adapt the existing nominal trajectories to new tasks during the training process.

## 3 Preliminaries

In this section, we describe the problem statement and provide fundamentals of some key techniques utilized in our adaptive robotic imitation framework.

### 3.1 Problem statement

Let A be a complex contact-rich insertion task *class*, which represents a set of tasks with topologically similar trajectories. We define a task $A_{\left(n\right)}\in A$ as the *nth* *instance* of A . **P**_(*n*)_ is the demonstrated trajectory profile of *A*_(*n*)_ consisting of *k* demonstrated trajectories, Γ, i.e., $P_{\left(n\right)}={\left\{\Gamma^{1},\Gamma^{2},\ldots ,\Gamma^{k}\right\}}_{\left(n\right)}$ , and each Γ in **P**_(*n*)_ consists of a sequence of the EEF poses, **p**, in the task space. Using the hybrid trajectory and force learning framework proposed by Wang et al. (2021), we can learn a proper control policy for each *A*_(*n*)_ if **P**_(*n*)_ is accessible.

**FIGURE 2:**
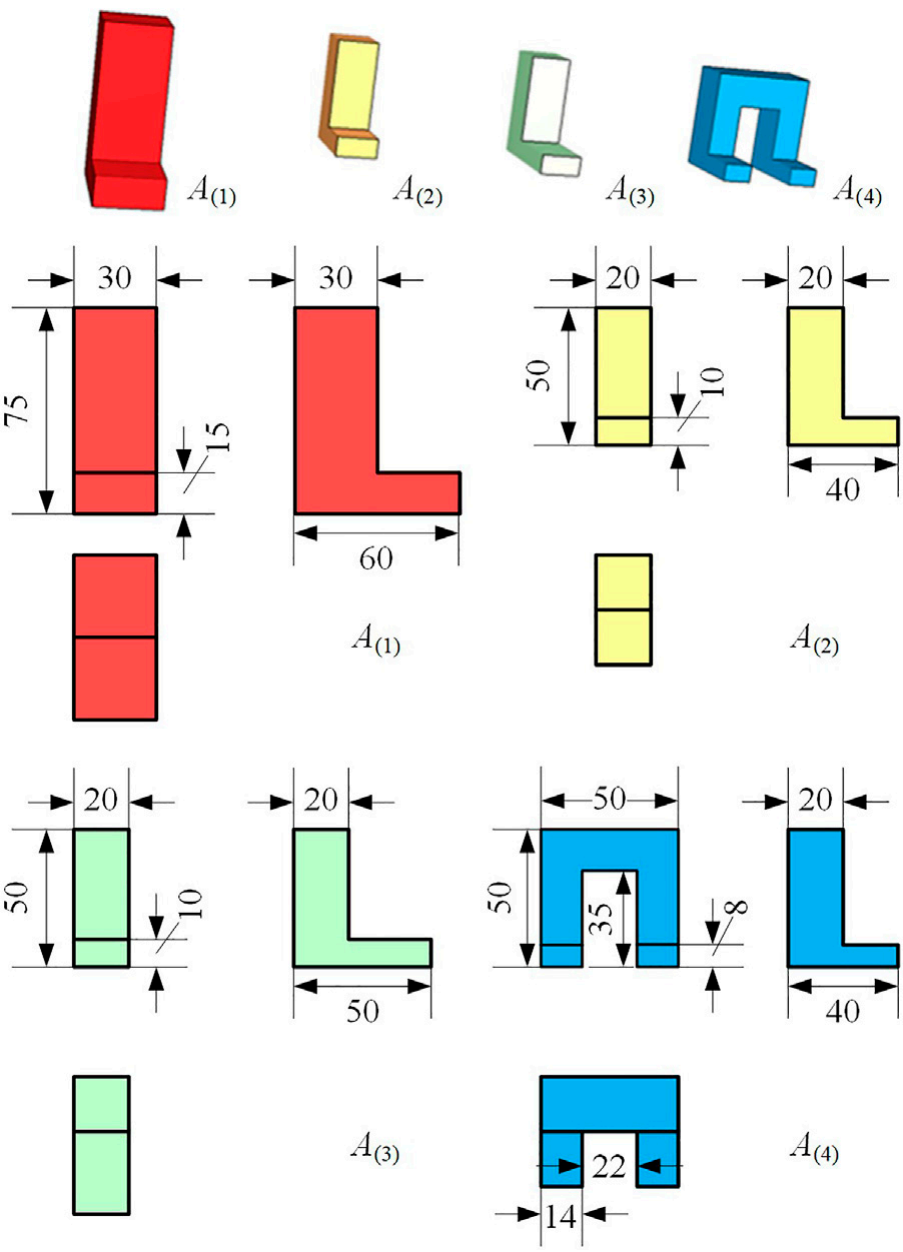
A class of L-shaped object insertion tasks. The shapes and sizes of workpieces are different among tasks, but these tasks possess topologically similar insertion trajectories (unit: mm).

To clarify, we assume an L-shaped object insertion (*L insertion*) task class, referred to as A . The goal of *L insertion* is to insert an L-shaped workpiece held by a robotic gripper into a groove with a corresponding shape, and the clearances are no more than 1 mm. There are some instances where $\left\{A_{\left(1\right)},A_{\left(2\right)},A_{\left(3\right)},A_{\left(4\right)}\right\}\in A$, and Figure 2 shows the L-shaped workpiece, **L**, involved in each instance. With *A*_(1)_ as the base instance, the **L** of *A*_(2)_ gets its shape by applying an affine transformation to the **L** of *A*_(1)_; the **L**s of *A*_(3)_ and *A*_(4)_ further reshape it by extending the bottom and doubling the entity, respectively.

**FIGURE 3:**
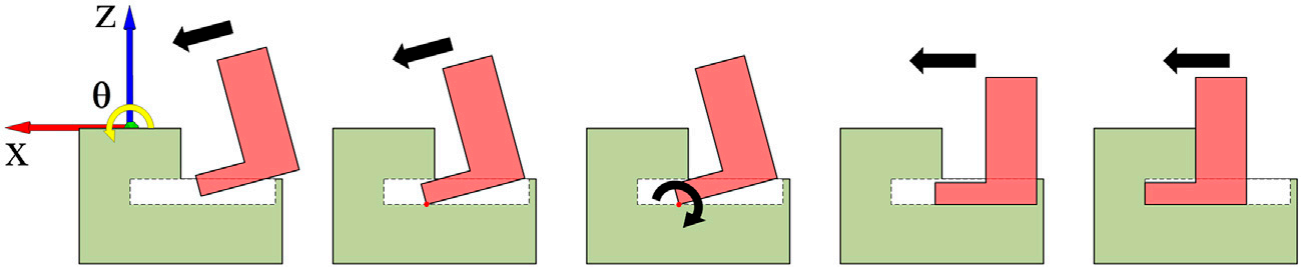
The insertion trajectory of *A*_(1)_.

In this paper, we assume that only the demonstrated trajectory profile of *A*_(1)_, **P**_(1)_, is available as shown in Figure 3. We know that other instances of A have similar trajectories to *A*_(1)_ but have no access to concrete information of these trajectories or geometric characteristics of objects involved in these instances. Although we can collect their trajectory profiles through demonstrations, it would be time-consuming and tedious when the number of instances is quite large, which brings huge burdens to the human demonstrator. Therefore, we need an effective trajectory learning approach that can adapt an existing trajectory profile to new similar scenarios to reduce the human burden, and this is the motivation that we introduce the DMPs to the hybrid trajectory and force learning framework.

### 3.2 Dynamical movement primitives

#### 3.2.1 Positional dynamical movement primitives

Following the modified formulation of positional DMP introduced by (Park et al., 2008), the differential equation of a one-dimensional positional DMP has three components. The first component is the transformation system that creates the trajectory plan:

$$\tau \dot{v}=K\left[\left(g-x\right)-\left(g-x_{0}\right)s+f\left(s\right)\right]-Dv$$ (1)

where x∈R and $v=\tau \dot{x}$ are the position and velocity of a prescribed point of the system, respectively. $\tau \in R^{+}$ is a temporal scaling factor. $x_{0},g\in R$ are the initial and goal positions, respectively. $K,D\in R^{+}$ are the spring and damping terms, respectively, and *D* is chosen as $D=2\sqrt{K}$ to keep the system critically damped. *s* is a phase variable, and it is governed by the second component of DMP formulation, a canonical system: $\tau \dot{s}=-\alpha s,\;\alpha \in R^{+}$ .

The third component is a nonlinear function approximation term (called forcing term), *f*, to shape the attractor landscape,

$$f\left(s\right)=\frac{\sum_{i=1}^{N}\omega_{i}\psi_{i}\left(s\right)}{\sum_{i=1}^{N}\psi_{i}\left(s\right)}s$$ (2)

where $\psi_{i}\left(s\right)=\exp\left(-h_{i}{\left(s-c_{i}\right)}^{2}\right)$ are Gaussian basis functions with centers *c*_*i*_ and widths *h*_*i*_, and *ω*_*i*_ is their weights.

In this paper, we utilize three-dimensional DMPs for the three positional degrees of freedom (DoF). Therefore, we rewrite Eq. 1 in multidimensional form as shown in Eq. 3:

$$\left\{\begin{array}{c} \tau \dot{v}=K\left[\left(g-x\right)-\left(g-x_{0}\right)s+f\left(s\right)\right]-Dv\;\\ \tau \dot{x}=v\; \end{array}\right.$$ (3)

Each DoF has its own transformation system and forcing term but shares the same canonical system.

#### 3.2.2 Orientational dynamical movement primitives

Besides positional DMPs, insertion tasks are also highly dependent on orientation. Therefore, we also utilize orientational DMPs (Pastor et al., 2011; Ude et al., 2014). A unit quaternion **q** ∈ *S* ^3^ is commonly used to describe an orientation because it provides a singularity-free and nonminimal representation of the orientation (Ude et al., 2014). *S* ^3^ is a unit sphere in $R^{4}$ . The transformation system of orientational DMPs is:

$$\left\{\begin{array}{c} \tau \dot{\eta }=K\left[2\log\left(g*\bar{q}\right)\right]-D\eta +f\left(s\right)\;\\ \tau \dot{q}=\frac{1}{2}\tilde{\eta }*q\; \end{array}\right.$$ (4)

where **g** ∈ *S* ^3^ denotes the goal quaternion orientation, $\bar{q}$ denotes the quaternion conjugation of **q**, and * denotes the quaternion product. $\tilde{\eta }={\left[0,\eta^{T}\right]}^{T}$ is the angular velocity quaternion. $K,D\in R^{3\times 3}$ are angular stiffness and damping gains, respectively. The canonical system and the nonlinear forcing term, **f**(*s*), are defined in the same way as the positional DMPs. We also use the quaternion logarithm log(⋅) and exponential map exp(⋅) as given in Ude et al. (2014).

#### 3.2.3 Coupling term

Eqs. 3 and 4 can be used to imitate a demonstrated trajectory. However, we sometimes desire to modify the behavior of the system online in practice. To modify a DMP online, an optional coupling term, *C*_*t*_, is usually added to the transformation system of DMP. For example, a one-dimensional positional DMP with *C*_*t*_ has the formulation as follows:

$$\tau \dot{v}=K\left(g-x\right)-Dv-K\left(g-x_{0}\right)+Kf\left(s\right)+\underline{C_{t}}$$ (5)

Ideally, *C*_*t*_ would be zero unless a special sensory event requires modifying the DMP. In the field of robotic manipulation, coupling terms have been used to avoid obstacles (Rai et al., 2014), to avoid joint limits (Gams et al., 2009), to grasp under uncertainty (Pastor et al., 2009), etc. This term is vital for our adaptive framework, and we will discuss it in the *Adaptive robotic imitation framework* section.

### 3.3 Goal-conditioned imitation learning

In a typical IL setting, the *ith* demonstrated trajectory Γ^*i*^ in a trajectory profile **P** is in the form of state–action pairs, i.e., $\Gamma^{i}=\left(s_{0}^{i},a_{0}^{i},\ldots ,s_{T}^{i},a_{T}^{i}\right)$ , where *T* represents the total time steps. For a complex nonlinear trajectory, some specific states, commonly known as bottleneck states, need to be reached to correctly imitate the whole trajectory. It is challenging for behavior cloning (BC), a conventional approach, which learns a policy *π*(**a**|**s**) from the state–action pairs, to imitate, such a trajectory due to compounding errors in the Markov decision process (MDP). Goal-conditioned IL (GCIL) is a self-supervised method that learns a goal-conditioned policy that has been proven to be more effective than BC in reproducing the said complex trajectory (Kaelbling, 1993; Schaul et al., 2015; Ding et al., 2019). In a goal-conditioned setting, the state–action pairs are replaced by state–action–goal triplets, $\left(s_{t}^{i},a_{t}^{i},s_{g}^{i}\right)$ , and a goal-conditioned policy *π*(**a**|**s**, **s**_*g*_), which attempts to match different goals is learned instead of *π*(**a**|**s**). Data relabeling (Lynch et al., 2019) is an effective data augmentation method usually used by GCIL, which treats each state $s_{t+k}^{i}$ visited within a demonstrated trajectory from $s_{t}^{i}$ to $s_{g}^{i}$ as a latent goal state. This technique is particularly effective in the low data regime where a few demonstrations are available.

Algorithm 1 | Modular learning process.

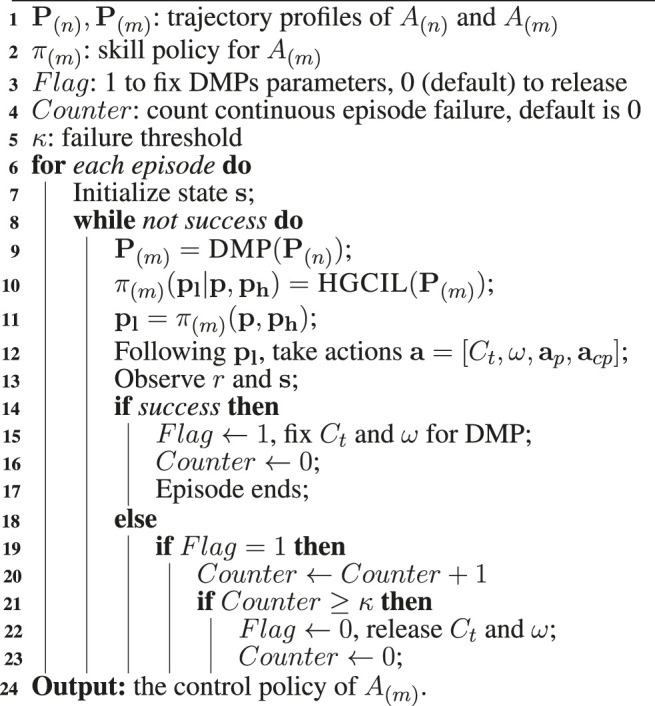


## 4 Adaptive robotic imitation framework

### 4.1 System overview

The architecture of our framework is shown in Figure 1, which is built on a hybrid trajectory and force learning framework from our previous work (Wang et al., 2021). It consists of a trajectory learning part and a force learning part. The former takes an existing trajectory profile, **P**_(*m*)_, of the task $A_{\left(m\right)}\in A$ as input and generates the nominal trajectory, $\Gamma_{\left(n\right)}^{N}$ , of another task $A_{\left(n\right)}\in A$ . $\Gamma_{\left(n\right)}^{N}$ is learned from **P**_(*m*)_ by an IL agent, which consists of an adaptive DMP module (ADMP) and a skill policy module. The force learning part is composed of an RL agent and a parallel position/force controller (Chiaverini and Sciavicco, 1993). The RL agent learns both the parameters and the position/orientation commands of the controller following $\Gamma_{\left(n\right)}^{N}$ to control the industrial rigid manipulator to finish *A*_(*n*)_ with proper force control policy. In the rest of this section, we will introduce each part of this framework in detail.

### 4.2 Modular learning strategy

In the proposed framework, we use a modular learning strategy because end-to-end learning can become very inefficient and even fail as networks grow (Glasmachers, 2017), which is known as the curse of dimensionality. In contrast, structured training of separate modules may be more robust. Moreover, assembly tasks are naturally divided into different subtasks that can be learned in different modules, e.g., in our problem setting, a task can be divided into a trajectory learning part and a force learning part. Therefore, we introduce DMPs into the framework in a modular learning fashion expecting to overcome the curse of dimensionality.

ADMP works in the trajectory learning part. It keeps constant after finding a *seemingly* suitable nominal trajectory $\Gamma_{\left(n\right)}^{N}$ of *A*_(*n*)_ with a small amount of trial and error, and then the framework only updates the parameters of the controller for the force learning at each training step. If the learning performance is constantly poor with the current $\Gamma_{\left(n\right)}^{N}$ after certain steps, ADMP will be updated again with a given frequency to search for an alternative $\Gamma_{\left(n\right)}^{N}$ . This mechanism is represented by the switch symbol in Figure 1. The whole modular learning process is shown in Algorithm 1.

### 4.3 Trajectory learning

#### 4.3.1 Adaptive action of adaptive dynamical movement primitives

In the trajectory learning, we hope to adapt trajectories in an existing task trajectory profile to new trajectories that are suitable for other similar tasks. Therefore, we introduce ADMP to achieve this goal. As we only use ADMP to realize spatial scaling, we set the temporal scaling factor *τ* to 1 in Eqs. 3 and 4.

As mentioned in the *Coupling term* section, the behaviors of ADMP can be modified by changing the coupling terms, *C*_*t*_, in Eq. 5. Therefore, it is a promising approach to learn proper *C*_*t*_ for ADMP to adapt to new scenarios. Moreover, the forcing term weights, *ω*, can also affect the resulting trajectories.

**FIGURE 4:**
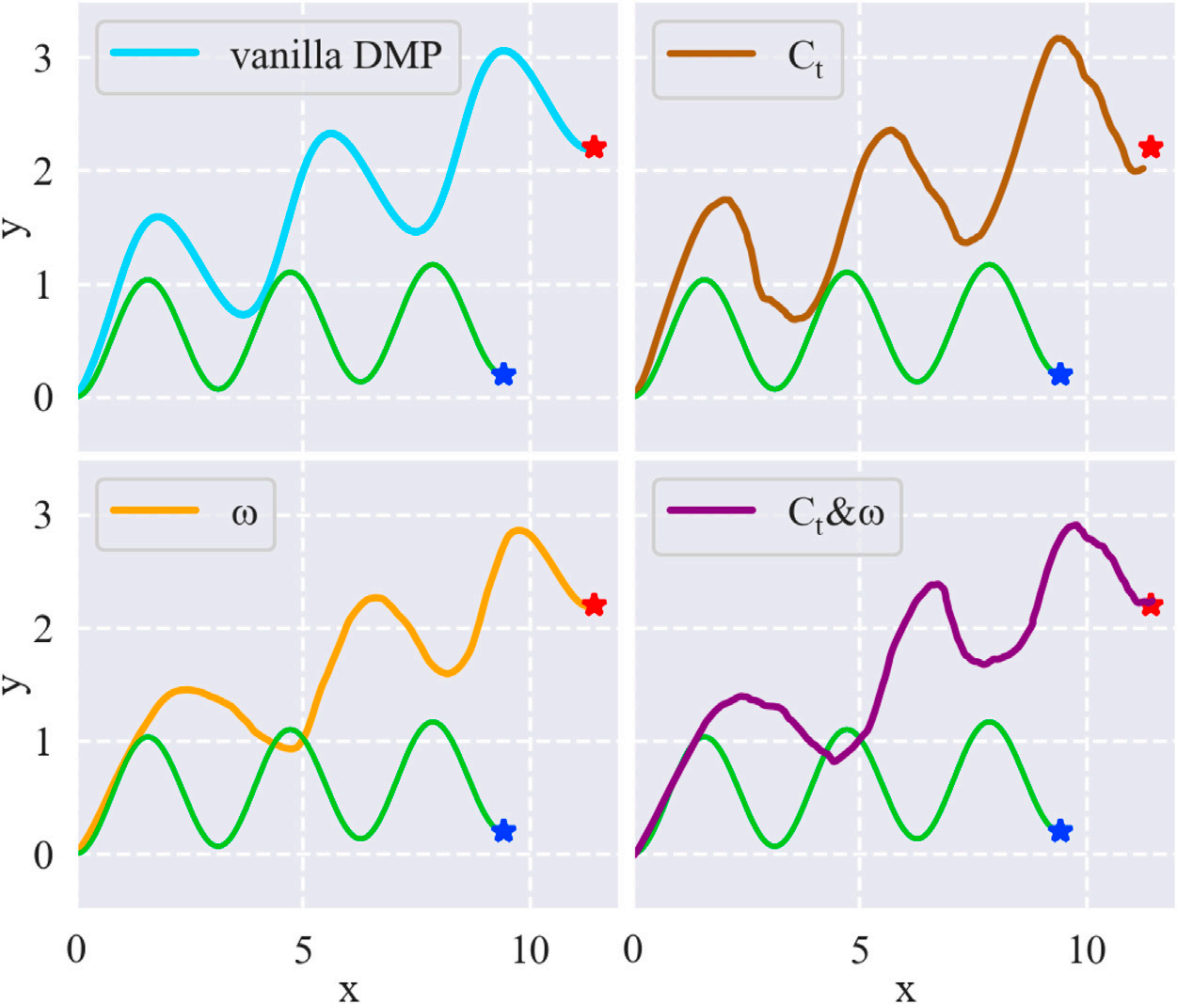
Comparison of different resulting trajectories by changing different components of the DMPs formulation. Green line is the original sine wave trajectory. Blue star and red star are the original goal and the new goal, respectively.

To discern how different components of the DMP formulation affect the results, we make an investigation by introducing random *C*_*t*_ or adding random noise to *ω* in the DMP formulation of a sine wave as depicted in Figure 4. The green line is a sine wave trajectory. We spatially scale the sine wave to match a new goal using two-dimensional DMPs. The first subfigure in Figure 4 shows the scaled trajectory using vanilla DMPs, which means no *C*_*t*_ is added, and *ω* is chosen to match the original trajectory without noise. With such a baseline, we then add 1) only *C*_*t*_; 2) only *ω* noise; and 3) both *C*_*t*_ and *ω* noise to the DMP formulation to observe the effects on the resulting trajectories. The results in Figure 4 indicate that 1) *C*_*t*_ facilitate local exploration based on the original trajectory, 2) noise added to *ω* leads to locally smooth but globally different trajectory, and 3) adding both *C*_*t*_ and *ω* results in a trajectory with both global shape change and local exploration.

Considering our requirements, the global change in trajectory may benefit the coarse adaptation to geometric characteristics of new workpieces, and the local exploration can help to tackle some delicate bottleneck states along the trajectory. Therefore, we choose to add both *C*_*t*_ and *ω* noise to the DMP formulation. In the framework, instead of meaningless variables, *C*_*t*_ and *ω* noise are learned by the RL agent through interacting with the environment.

#### 4.3.2 Hierarchical goal-conditioned imitation learning

We train the skill policy using an HGCIL approach proposed in our previous work (Wang et al., 2021). Following the goal-conditioned setting in the *Goal-conditioned imitation learning* section, we reorganize the original trajectory profile **P**_(*n*)_ into a hierarchical goal-conditioned (HGC) trajectory profile $P_{\left(n\right)}^{skill}$ . A trajectory $\Gamma \in P_{\left(n\right)}^{skill}$ consists of a sequence of poses (**p**_0_, **p**_1_, … , **p**_*T*_), which are Cartesian poses of EEF in our framework. Sliding along each sequence in **P**_(*n*)_ with two predefined hierarchical windows, *W*_*s*_ and *W*_*m*_, we obtain a new sequence of triplets,

$$\begin{array}{l} \left(p,p_{l},p_{h}\right)=\left(p_{t},p_{t+\min\left(w,W_{m}\right)},p_{t+w}\right),if\;t+w\leq T\\ t=1,2,\ldots ,T;w=1,2,\ldots ,W_{s}. \end{array}$$

where **p**, **p**_*l*_, and **p**_*h*_ represent the current pose, the subgoal pose, and the goal pose, respectively. Note that **p**_*l*_ plays the role of action between two consecutive states here. All these triplets compose $P_{\left(n\right)}^{skill}$ and the skill policy *π*(**p**_*l*_|**p**, **p**_*h*_) is trained using a fully connected neural network with three hidden layers, each with 256 units, a dropout rate of 0.1, and ReLu as the activation function, which maps the observation, (**p**, **p**_*h*_), to the action, **p**_*l*_. With the skill policy *π*(**p**_*l*_|**p**, **p**_*h*_), the IL agent can spontaneously find subgoals, **p**_*l*_, for a distant goal along the trajectory and provides **p**_*l*_ to the parallel controller. All these subgoals compose the nominal trajectory, $\Gamma_{\left(n\right)}^{N}$ . Since **p**_*l*_ can be periodically updated based on **p** and **p**_*h*_, the motion drift of EEF is constrained, and the goal-conditioned setting assists the EEF in recovering from unseen states.

### 4.4 Force learning

#### 4.4.1 Reinforcement learning-based controller

An RL-based controller proposed in our previous work (Beltran-Hernandez et al., 2020) is responsible for learning the proper force control policy decided by the gain parameters of the controller, **a**_*cp*_, as well as the position/orientation commands of EEF, **a**_*p*_. The RL-based controller consists of an RL agent and a parallel position/force controller. The parallel position/force controller includes a proportional derivative (PD) controller generating part of the movement command, $p_{c}^{p}$ , based on the position feedback, and a proportional integral (PI) controller adjusting the movement command by $p_{c}^{f}$ according to the force feedback.

The learning process starts with **p**_*l*_ from the trajectory learning every time step. **p** is the actual Cartesian pose of EEF, and **f** = [ ***f***, ***τ***] is the contact force, where $f\in R^{3}$ is the force vector and $\tau \in R^{3}$ is the torque vector. **f**_*g*_ is the reference force of the insertion task. The pose error of EEF, **p**_*e*_ = **p**_*l*_ − **p**, the velocity of the EEF, $\dot{p}$ , and **f** serve as inputs to the RL agent, while **p**_*e*_ and **f** also serve as feedback to the parallel position/force controller. For the controller, the RL agent gives policy actions consisting of **a**_*p*_ and **a**_*cp*_. **a**_*p*_ = [***v***, ***w***] are the position/orientation commands where $v\in R^{3}$ is the position and $w\in R^{4}$ is the quaternion to control the movements of the robot; $a_{cp}=\left[K_{p}^{p},K_{p}^{f},S\right]$ are the gain parameters of the controller where $K_{p}^{p}$ , $K_{d}^{p}=2\sqrt{K_{p}^{p}}$ , $K_{p}^{f}$ , and $K_{i}^{f}=0.01K_{p}^{f}$ are PD proportional, PD derivative, PI proportional, and PI integral gains, respectively, and

$$S=\mathrm{diag}\left(s_{1},s_{2},s_{3},s_{4},s_{5},s_{6}\right),\;s_{n}\in \left[0,1\right]$$ (6)

is the selection matrix, whose elements correspond to the degree of control that each controller has over a given direction. Finally, the actual position command, $p_{c}=a_{p}+p_{c}^{p}+p_{c}^{f}$ , is produced by the controller based on all inputs and sent to the manipulator.

#### 4.4.2 Algorithm and reward

We use Soft-Actor-Critic (SAC) (Haarnoja et al., 2018) as the RL algorithm of the scheme, which is a state-of-the-art model-free and off-policy actor-critic deep RL algorithm based on the maximum entropy RL framework. It encourages exploration according to a temperature parameter, and the core idea is to succeed in the task while acting as randomly as possible. As an off-policy algorithm, it can use a replay buffer to reuse information from recent operations for sample-efficient training. We use a reward function as follows:

$$r\left(s\right)=w_{1}M\left({\left\|\frac{p_{e}}{p_{\max}}\right\|}_{1,2}\right)+w_{2}M\left({\left\|\frac{f_{e}}{f_{\max}}\right\|}_{2}\right)+\gamma .$$ (7)

**f**_*e*_ = **f**_*g*_ − **f** is the contact force error. **p**_*max*_ and **f**_*max*_ are defined maximum values. *y* = *M*(*x*), *x* ∈ [1, 0] linearly maps *x* to *y* ∈ [1, 0]. Therefore, the smaller **p**_*e*_ and **f**_*e*_ are, the higher the reward is. ${\left\|z\right\|}_{1,2}$ is the *l*_12_ norm (Levine et al., 2016), which is given by $\frac{1}{2}{\left\|z\right\|}^{2}+\sqrt{\alpha +z^{2}}$ . This norm is used to encourage the EEF to precisely reach the target position, but to also receive a larger penalty when far away. *γ* is the auxiliary term, which can be a positive reward (100) for finishing the task successfully, a negative one (−50) for excessive force, or 0 otherwise. *w*_1_ and *w*_2_ are hyperparameters to weight the components.

## 5 Experimental evaluation

In this section, we evaluate the efficacy of our adaptive robotic imitation framework in learning a class of complex contact-rich insertion tasks from a single instance. We perform a sequence of empirical evaluations using the *L insertion* task class. We divide this section into three parts: first, applying the framework on a simulated environment to study its sample efficiency, generalizability to different task instances, and safety during the training sessions; second, applying the framework to real insertion tasks to further validate its adaptiveness in the physical world; and third, ablation studies to investigate the effect of different components on the overall performance of our framework.

### 5.1 Implementation details

**FIGURE 5:**
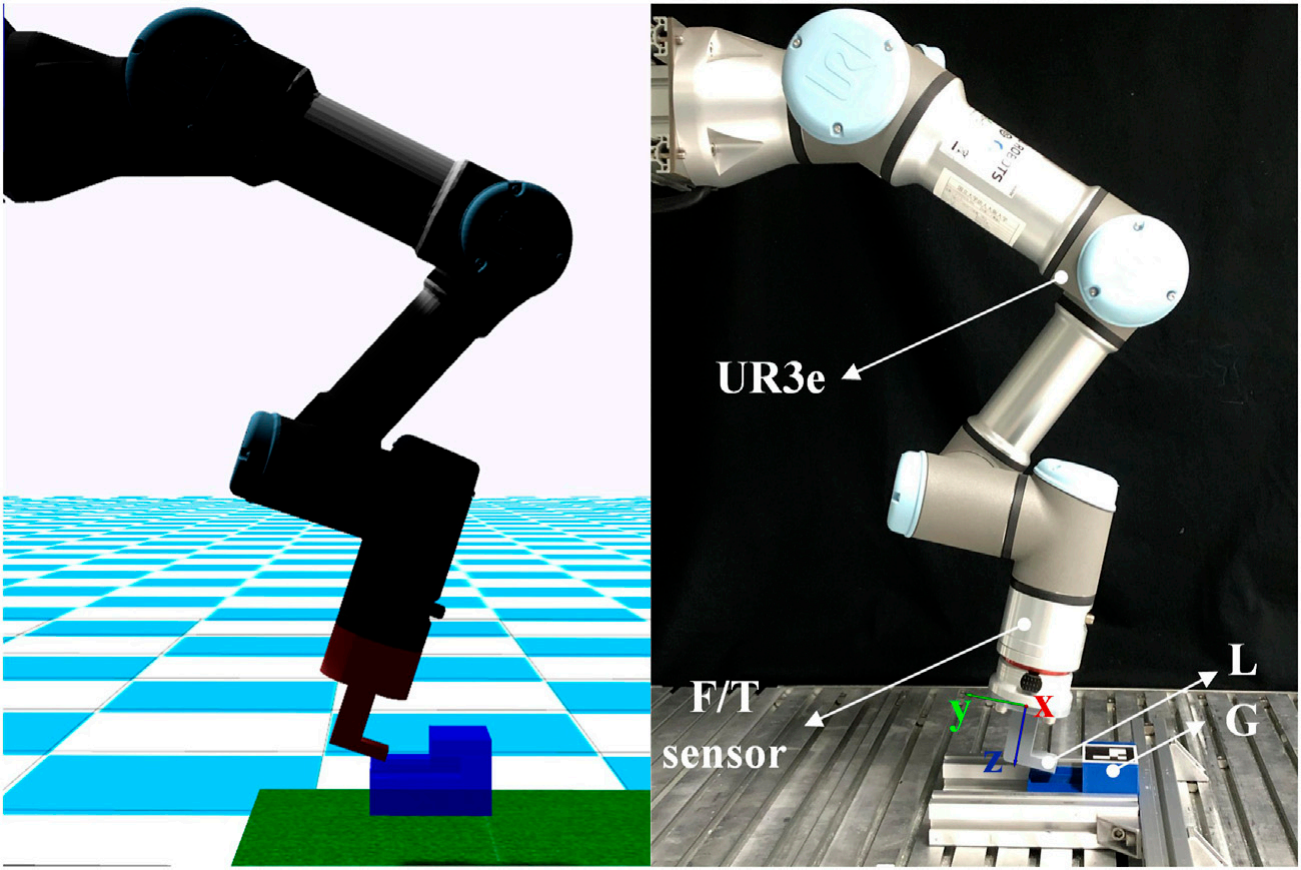
The simulated environment in Gazebo and the real experiment environment with a UR3e robotic arm. We show the setup for *A*_(2)_ task where L is directly attached to the robot.

We evaluate the proposed framework both on a simulated environment built in the Gazebo nine and on a real UR3e robotic arm as shown in Figure 5. The real UR3e robotic arm uses a control frequency of 500 Hz, which is the maximum available for the robot. The RL control policy runs at a frequency of 20 Hz on both the simulated environment and the real robot. The training sessions are performed on a computer with a GeForce RTX 2060 SUPER GPU and an Intel Core i7-9700 CPU. The implementation of the ADMP method was based on the DMP implementation from the DMP++ (Ginesi et al., 2019) repository, and for the RL agent, we used the SAC implementation from the TF2RL (Ota, 2020) repository.

### 5.2 Evaluation on simulated environment

First, we evaluated the efficacy of the adaptive robotic imitation framework on the simulated environment. We used the *L insertion* task class described in the *Problem statement* section, and we assumed access to only a trajectory profile of *A*_(1)_ consisting of six demonstrated trajectories.

#### 5.2.1 Sample efficiency

The most concerning point of the learning framework is the sample efficiency. By providing a nominal trajectory learned from demonstration to the RL learning process, the sample efficiency can be largely improved according to our previous work (Wang et al., 2021). However, the framework in this paper indirectly generates the nominal trajectory by adapting existing trajectories using ADMP and may cost more time than using demonstrated trajectories. Therefore, we are interested in whether the framework is still sample-efficient compared with other alternatives.

**FIGURE 6:**
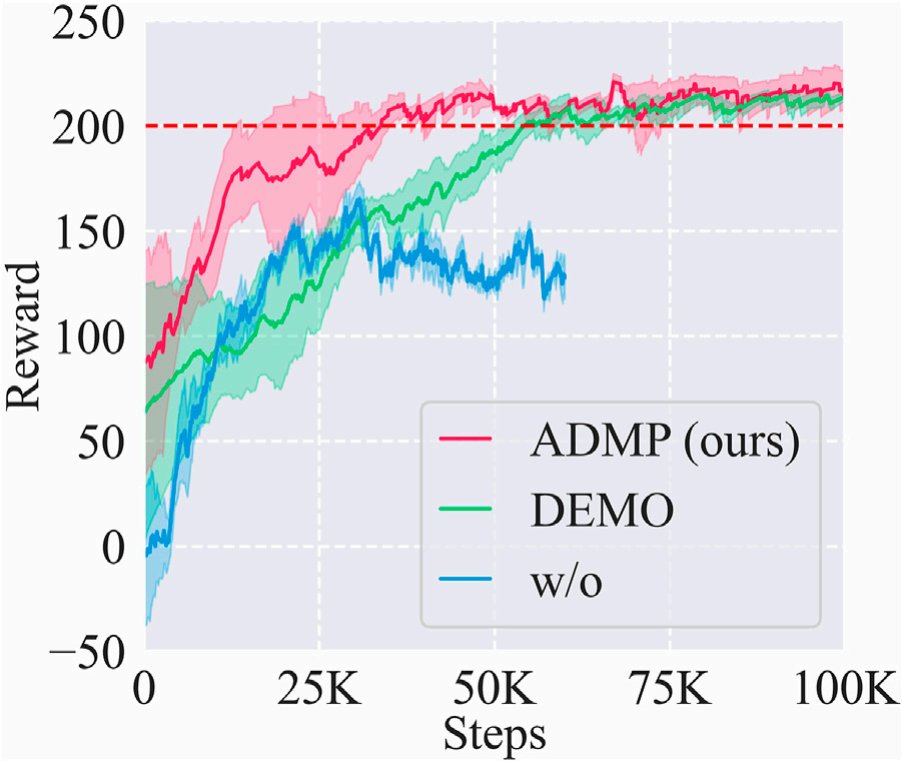
Learning curves of the training sessions on *A*_(2)_ task with frameworks using different trajectory learning methods: Adaptive DMPs (ADMP), demonstrated trajectory (DEMO), and RL without trajectory learning (w/o). The red dashed line represents the near-optimal reward.

We compared the learning curves of training sessions on *A*_(2)_ task with frameworks using different trajectory learning methods: ADMP (ours), demonstrated trajectory (DEMO), and RL from scratch (w/o) as shown in Figure 6.

Among these methods, ADMP showed the highest sample efficiency of 40 K steps, even higher than the baseline DEMO (55 K), and the learning result of ADMP was also as good as the DEMO. Although the better performance of ADMP than DEMO may result from suboptimal demonstration, this result indicated that introducing the DMP component into our framework was indeed effective in adapting to new tasks and alleviating human demonstration burden, and the sample efficiency was at least not lower than using demonstrated trajectories of new tasks.

#### 5.2.2 Generalizability

**FIGURE 7:**
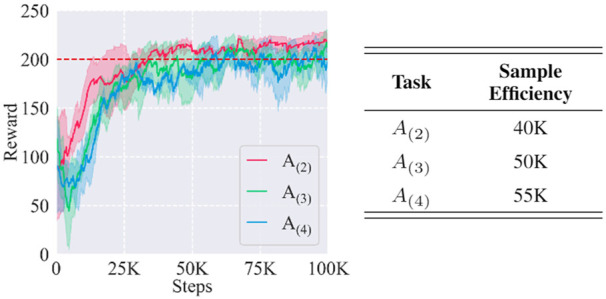
Learning curves and sample efficiencies of task instances *A*_(2)_, *A*_(3)_, and *A*_(4)_. The red dashed line represents the near-optimal reward.

Since the proposed framework displayed good adaptation to *A*_(2)_ task, we then tested with *A*_(3)_ and *A*_(4)_ to study its generalizability to different tasks. The result is shown in Figure 7. It indicated that the framework could generalize among different kinds of task instances with good sample efficiencies and learning results. In detail, the steps cost for convergence in learning *A*_(2)_, *A*_(3)_, and *A*_(4)_ were 40, 50, and 55 K steps, respectively. We analyzed that different sample efficiencies mainly resulted from their different difficulties: the object shapes in *A*_(2)_ were the most similar to *A*_(1)_ with an affine transformation, while the other two involved more variations.

#### 5.2.3 Safety

**FIGURE 8:**
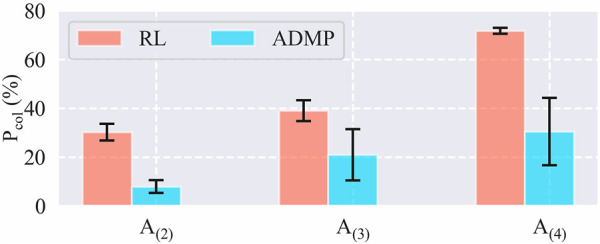
Collision percentage during the training sessions.

Finally, we compared the collision percentage during the training sessions of each task using frameworks with and without ADMP as shown in Figure 8. Five training sessions were implemented for each pair of task and framework, and the collision percentage of each training session, *P*_*col*_, is calculated by:

$$P_{col}=\frac{Total\;Collision\;Number}{Total\;Episode\;Number}\times 100\%.$$

With ADMP, the collision percentages of *A*_(2)_, *A*_(3)_, and *A*_(4)_, were diminished to 7.9%, 20.9%, and 30.5% from 30.2%, 39%, and 72.6%, respectively. The result indicated that the proposed framework with ADMP was also qualified for our requirement of lowering the chance of collision during the training sessions, which reduces the equipment wear and tear and the risk of damaging the workpieces on real hardware.

### 5.3 Experiments on a real robot

After evaluating the sample efficiency, generalizability, and safety of the framework on the simulated environment, we applied it to some real insertion tasks belonging to the *L insertion* task class to test its adaptiveness in the physical world.

#### 5.3.1 Sim-to-real transfer

**FIGURE 9:**
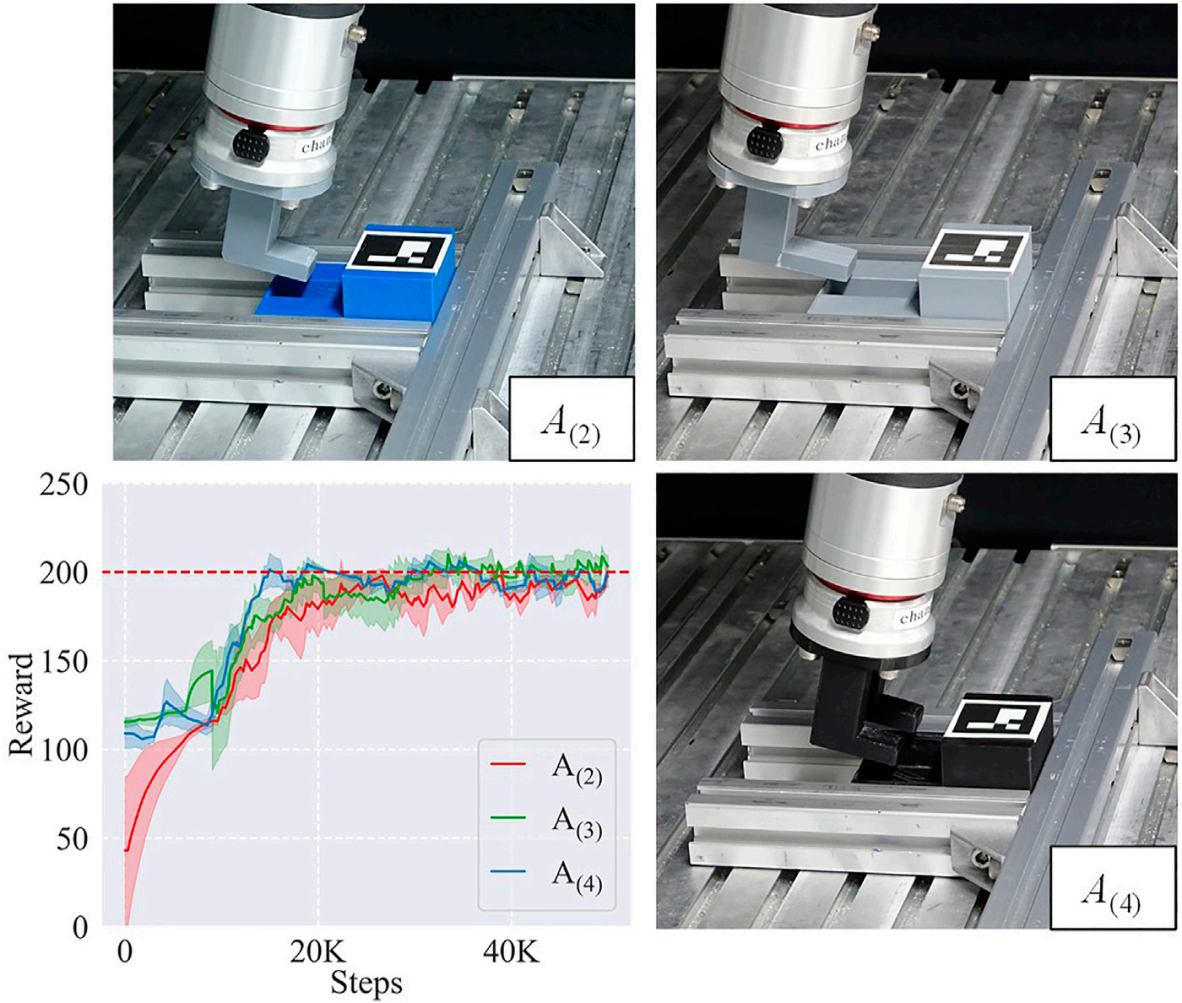
Sim-to-real tasks *A*_(2)_, *A*_(3)_, and *A*_(4)_ and their learning curves. The L objects are directly attached to the robot.

We first executed sim-to-real transfers using a trained IL agent, which learned the skill policies on simulation and obtained the control policies for *A*_(2)_, *A*_(3)_, and *A*_(4)_ on the real hardware. The L objects in these tasks were directly attached to the robot for stability. The learning curves are shown in Figure 9. Benefiting from the learned skill policies, our framework learned good control policies for *A*_(2)_, *A*_(3)_, and *A*_(4)_ at about 20 K steps. Although it took some time for the RL agent to adapt to the physical world, the result indicated that the skill policies learned by the framework on the simulation provided good initialization and effectively enhanced the sample efficiency of the real learning process.

#### 5.3.2 Real assembly tasks

**FIGURE 10:**
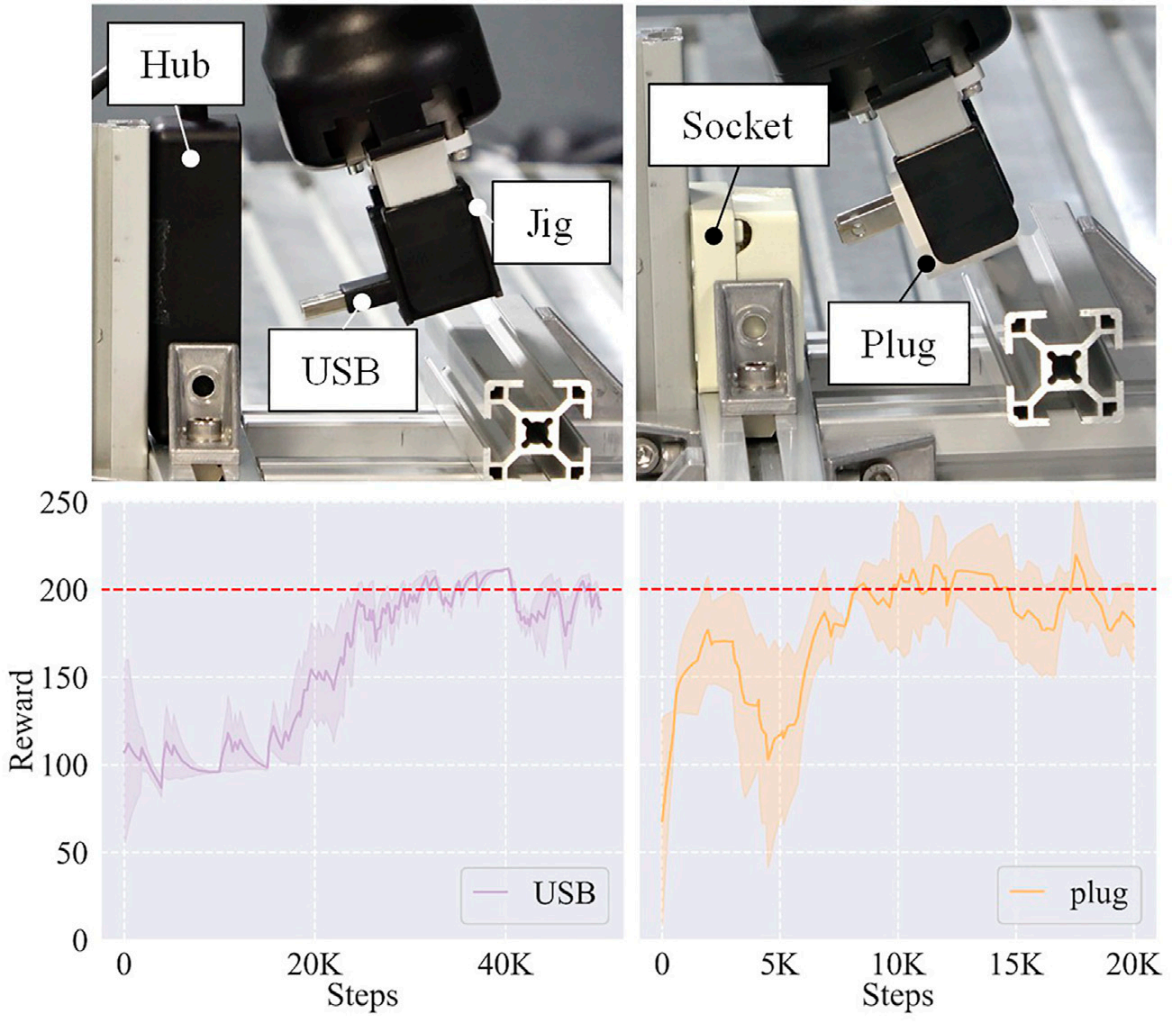
Two real assembly tasks and their learning curves. Left: USB insertion task. Right: plug insertion task. The objects are grasped by the gripper. A jig is used in the USB insertion to improve the stability considering the contact area between the USB and the gripper.

We then utilized two real assembly tasks, a USB insertion task and a plug insertion task, to further validate the generalizability of the framework. As shown in Figure 10, the USB and the plug were grasped by the gripper. We assumed that they were in stable poses so that their positions did not change too much during the training sessions. Considering the contact area, we added a jig between the USB and the gripper to improve the stability in case that the friction was not enough to resist the contact force. As for the plug, we did not utilize a jig. A structural aluminum profile played the role of an obstacle to prevent the USB or the plug from inserting into the hub or the socket directly in each task, so that the EEF had to adopt a trajectory like the *L insertion* to finish the task. Figure 10 also shows their learning curves. It took the framework about 30 K steps and 10 K steps to learn these two tasks, respectively. We analyzed that the difference between the sample efficiencies of different tasks resulted from their different trajectory proximity to the initial trajectory—the trajectory of the plug insertion was more similar to the original demonstrated trajectory of the *L insertion* so that the learning process was faster. Note that we utilized a jig in the USB insertion to reduce the slip between the object and the gripper but not in the plug insertion. This is why the performance dropped at about 3 K steps in the plug insertion while the performance of the USB insertion kept improving.

**FIGURE 11:**
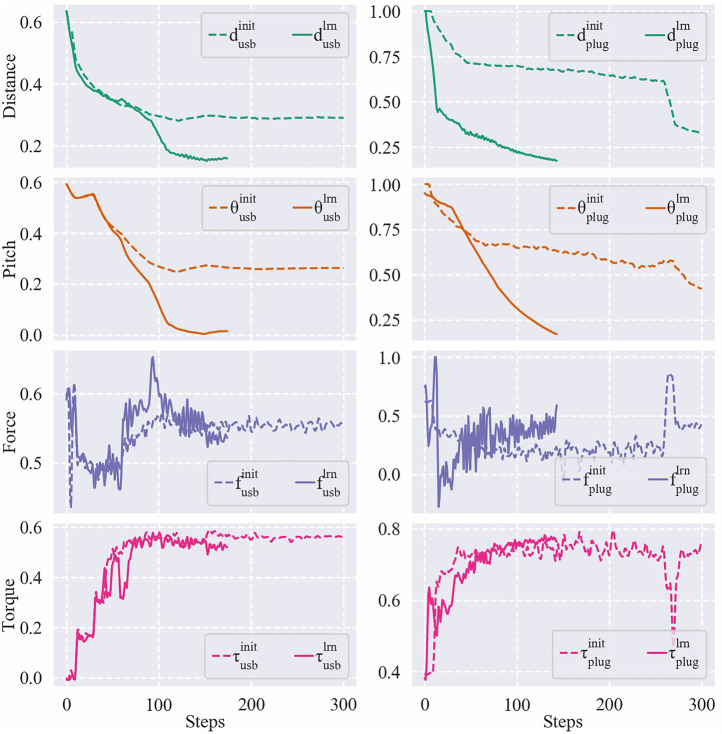
Distance errors, pitch angle errors, and force/torque data of a USB insertion **(left)** and a plug insertion **(right)** using their initial/learned policies. The error values have been mapped to a range of [1, 0] and the force/torque values have been mapped to a range of [−1, 1].

Table 1 displays the success rates and average steps cost among 20 trials for each task. In each trial, the EEF was initially set to a random pose in a distance range of [15, 45] (unit: mm) and a pitch angle range of [10, 30] (unit: °) away from the target pose. Figure 11 shows the results of the initial and the learned control policies of the two tasks, including the Euclidean distance errors of EEF, pitch angle errors, and the force/torque data during the evaluation process. Note that although only the result of a single run is provided for each policy, it is typical enough to verify the effectiveness of the proposed framework on learning good policies for the tasks.

**TABLE 1:** Performance on the two real assembly tasks.

Task	Metrics	Start pose			
		Pose 1	Pose 2	Pose 3	Pose 4
USB	Success rate	0.95	0.90	1.0	0.90
	Avg. steps	172.0	156.1	120.5	113.6
Plug	Success rate	1.0	1.0	0.90	0.90
	Avg. steps	89.3	176.7	131.2	317.8

### 5.4 Ablation studies

In this part, we executed two ablation studies to investigate how different hyperparameters and strategies affected the performance of the proposed framework. We ran each ablation study on *A*_(2)_ task following the settings in the *Evaluation on simulated environment* section.

#### 5.4.1 Effects of the dynamical movement primitive components

In the *Adaptive action of adaptive dynamical movement primitives* section, we provide a simple investigation on how *C*_*t*_ and *ω* of the DMPs affect the generalized trajectory, and the conclusion is that *C*_*t*_ benefits the local exploration while *ω* benefits the global change of the trajectory. As we assume that both the local exploration and the global change are necessary to efficiently learn the new trajectory, we tune both *C*_*t*_ and *ω* of the DMPs during the learning process.

**FIGURE 12:**
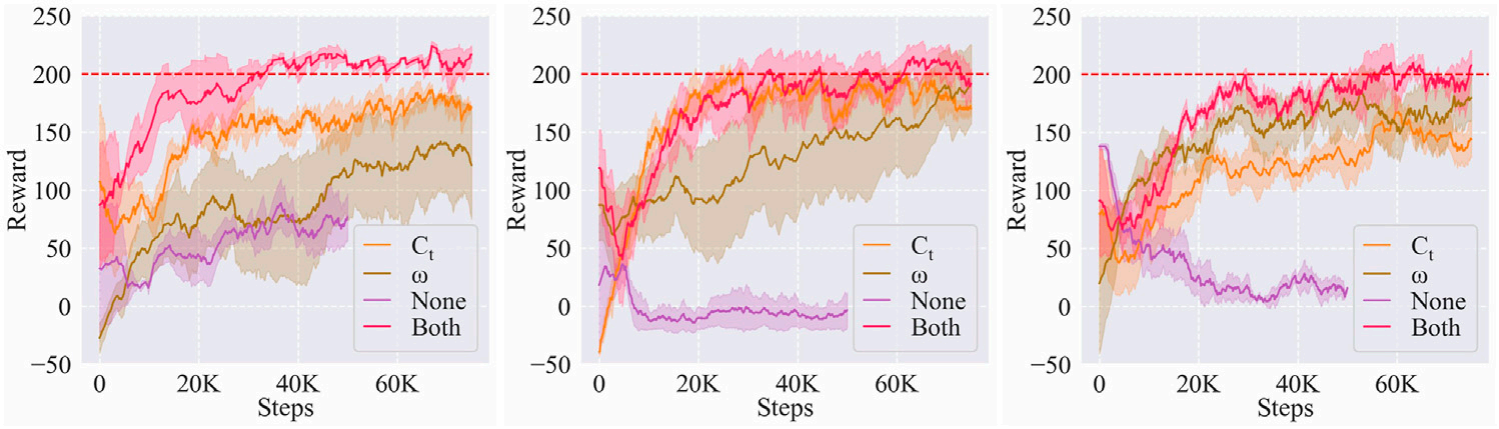
Effects of the DMPs components on the learning performance of *A*_(2)_ **(left)**, *A*_(3)_ **(middle)**, and *A*_(4)_ **(right)** tasks.

In this part, we investigated whether such a choice indeed improved the learning performance. We compared the learning performance of four choices: 1) ADMP (tuning both *C*_*t*_ and *ω*); 2) tuning *C*_*t*_; 3) tuning *ω*; 4) vanilla DMP (tuning neither *C*_*t*_ nor *ω*), on *A*_(2)_, *A*_(3)_, and *A*_(4)_ tasks. The learning curves are shown in Figure 12. The result showed that ADMP could guarantee both the learning speed and the stability on new tasks. Although separately tuning *C*_*t*_ or *ω* could also obtain good performances on some tasks, it depended on the tasks so that it was less universal than ADMP. Also, the vanilla DMPs hardly took effect without parameter tuning through RL, which meant that the intrinsic compliance of the controller could not tackle new tasks effectively.

#### 5.4.2 Effects of the number of demonstrated trajectories

**FIGURE 13:**
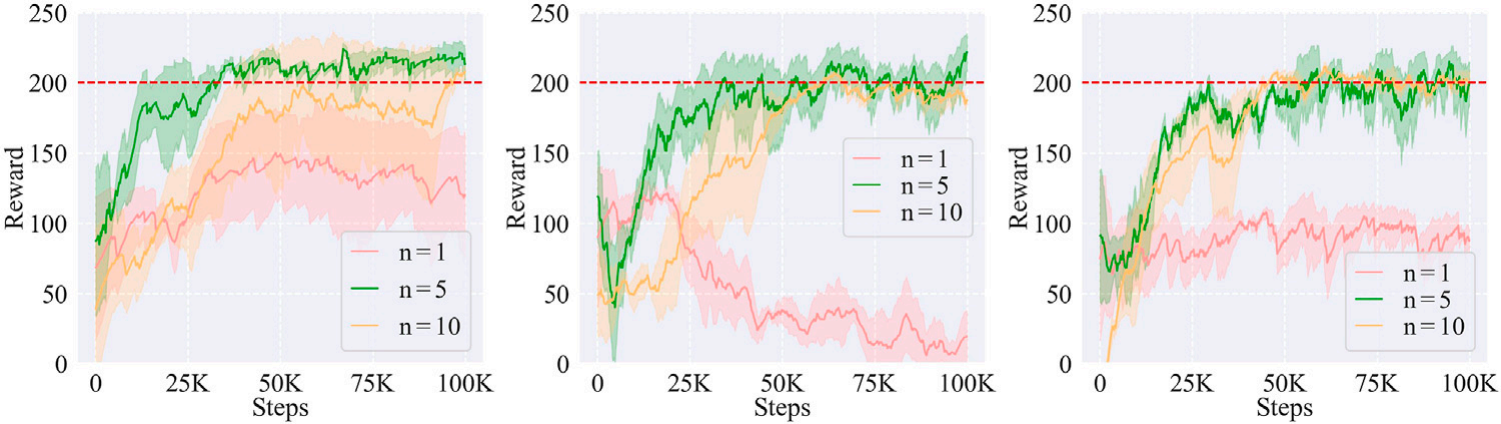
Effects of the number of demonstrated trajectories on the learning performance of *A*_(2)_ **(left)**, *A*_(3)_ **(middle)**, and *A*_(4)_ **(right)** tasks.

In our framework, the skill policy plays an important role to generate the nominal trajectory whose quality affects the learning performance. Therefore, we investigated how the number of demonstrated trajectories to train the skill policy would affect the learning results. We tested three numbers, *n* = 1, 5, 10, and plotted the results as shown in Figure 13.

When there was only a single trajectory, the learning performance was poor because it was difficult for the skill policy trained with limited data to handle unseen states during the learning process. However, when there were 10 trajectories, the large amount of data conversely confused the skill policy because of the high redundancy so that the performance was unstable. Therefore, we chose 5 as the optimal number of demonstrated trajectories, and all the results in the *Evaluation on simulated environment* and *Experiments on a real robot* sections 5-2 and 5-3 were produced using this number.

#### 5.4.3 Effects of the modular learning strategy

As mentioned in the *Modular learning strategy* section, we utilized a modular learning strategy for the learning of ADMP parameters assuming the curse of dimensionality would lower the performance of RL. Table 2 shows the number of parameters to tune in the learning process. First, following the parameter selection of Wang et al. (2021), we used six parameters for the position/orientation command, one $K_{p}^{p}$ parameter for the PD control, one $K_{p}^{f}$ parameter for the PI control, and six parameters for the selection matrix, **S**. Then, we assigned the coupling terms, *C*_*t*_, and the forcing term weights, *ω*, six parameters, respectively, which were used to adjust the trajectory in the six DoFs. Therefore, there were, in total, 26 parameters for different functional components involved in the learning process. Under the modular strategy in Algorithm 1, the number of parameters was reduced to 14 by fixing the 12 DMP parameters when a promising trajectory was found, which was assumed to be more robust than tuning all the 26 parameters simultaneously.

**TABLE 2:** Action space of the learning process.

Parameters	Pose	Controller			DMPs	
		PD	PI	S	*C*_*t*_	*ω*
Number	6	1	1	6	6	6

**FIGURE 14:**
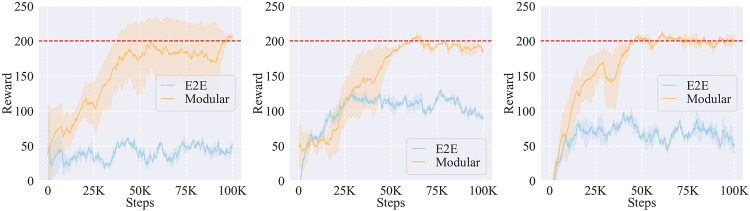
Effects of the modular learning and the end-to-end (E2E) learning on the learning performance of *A*_(2)_ **(left)**, *A*_(3)_ **(middle)**, and *A*_(4)_ **(right)** tasks.

To verify this assumption, we compared the modular learning with the end-to-end (E2E) learning as shown in Figure 14. We used 10 demonstrated trajectories for each task in this comparison. From the results, we found that it was hard for the E2E learning to converge, while the modular learning possessed relatively higher learning speed. It indicated that modular learning was more suitable for our framework than E2E learning when there were large numbers of parameters with different functions to tune.

## 6 Conclusion

In this work, we propose an adaptive robotic imitation framework for the hybrid trajectory and force learning of complex contact-rich insertion tasks. The framework is composed of learning the nominal trajectory through a combination of IL and RL, and learning the force control policy through an RL-based force controller. We highlight the use of the adaptive DMPs (ADMP), where the coupling terms and the weights of forcing terms in the DMP formulation are learned through RL to effectively adapt the trajectory profile of a single task to new tasks with topologically similar trajectories, which alleviates human repetitive demonstration burdens.

The experimental results show that the proposed framework is comparably sample efficient as a framework using explicitly demonstrated trajectories, has good generalizability among different instances in a task class, and is qualified for the safety requirement by lowering the chance of collision during the training sessions compared with the model-free RL approach. Moreover, the ablation studies show that a proper number of demonstrated trajectories and the modular learning strategy play vital roles in the proposed framework, which affects the speed and the stability of the learning process.

From the experimental results on the real hardware, we also found that the topological similarity of trajectories could affect the learning speed. Therefore, it may improve the efficacy of adapting the DMP parameters if we can represent new trajectories topologically close to the previous ones, and it remains an interesting issue for our future research.

## Data Availability

The raw data supporting the conclusion of this article will be made available by the authors, without undue reservation.
